# Serodiagnosis of *Echinococcus* spp. Infection: Explorative Selection of Diagnostic Antigens by Peptide Microarray

**DOI:** 10.1371/journal.pntd.0000771

**Published:** 2010-08-03

**Authors:** Claudia List, Weihong Qi, Eva Maag, Bruno Gottstein, Norbert Müller, Ingrid Felger

**Affiliations:** 1 Department of Medical Parasitology and Infection Biology, Swiss Tropical and Public Health Institute, Basel, Switzerland; 2 University of Basel, Basel, Switzerland; 3 Functional Genomics Center Zurich, ETH Zurich, Zurich, Switzerland; 4 Department of Medical and Diagnostic Services, Swiss Tropical and Public Health Institute, Basel, Switzerland; 5 Institute of Parasitology, University of Bern, Bern, Switzerland; Universidad Nacional Autónoma de México, Mexico

## Abstract

**Background:**

Production of native antigens for serodiagnosis of helminthic infections is laborious and hampered by batch-to-batch variation. For serodiagnosis of echinococcosis, especially cystic disease, most screening tests rely on crude or purified *Echinococcus granulosus* hydatid cyst fluid. To resolve limitations associated with native antigens in serological tests, the use of standardized and highly pure antigens produced by chemical synthesis offers considerable advantages, provided appropriate diagnostic sensitivity and specificity is achieved.

**Methodology/Principal Findings:**

Making use of the growing collection of genomic and proteomic data, we applied a set of bioinformatic selection criteria to a collection of protein sequences including conceptually translated nucleotide sequence data of two related tapeworms, *Echinococcus multilocularis* and *Echinococcus granulosus*. Our approach targeted alpha-helical coiled-coils and intrinsically unstructured regions of parasite proteins potentially exposed to the host immune system. From 6 proteins of *E. multilocularis* and 5 proteins of *E. granulosus*, 45 peptides between 24 and 30 amino acids in length were designed. These peptides were chemically synthesized, spotted on microarrays and screened for reactivity with sera from infected humans. Peptides reacting above the cut-off were validated in enzyme-linked immunosorbent assays (ELISA). Peptides identified failed to differentiate between *E. multilocularis* and *E. granulosus* infection. The peptide performing best reached 57% sensitivity and 94% specificity. This candidate derived from *Echinococcus multilocularis* antigen B8/1 and showed strong reactivity to sera from patients infected either with *E. multilocularis* or *E. granulosus*.

**Conclusions/Significance:**

This study provides proof of principle for the discovery of diagnostically relevant peptides by bioinformatic selection complemented with screening on a high-throughput microarray platform. Our data showed that a single peptide cannot provide sufficient diagnostic sensitivity whereas pooling several peptide antigens improved sensitivity; thus combinations of several peptides may lead the way to new diagnostic tests that replace, or at least complement conventional immunodiagnosis of echinococcosis. Our strategy could prove useful for diagnostic developments in other pathogens.

## Author Summary

Crude or purified, somatic or metabolic extracts of native antigens are routinely used for the serodiagnosis of human helminthic infections. These antigens are often cross-reactive, i.e., recognized by sera from patients infected with heterologous helminth species. To overcome limitations in antigen production, test sensitivity and specificity, chemically synthesized peptides offer a pure and standardized alternative, provided they yield acceptable operative characteristics. Ongoing genome and proteome work create new resources for the identification of antigens. Making use of the growing amount of genomic and proteomic data available in public databases, we tested a bioinformatic procedure for the selection of potentially antigenic peptides from a collection of protein sequences including conceptually translated nucleotide sequence data of *Echinococcus multilocularis and E. granulosus* (Plathyhelminthes, Cestoda). The *in silico* selection was combined with high-throughput screening of peptides on microarray and systematic validation of reactive candidates in enzyme-linked immunosorbent assay. Our study proved the applicability of this approach for selection of peptide antigens with good diagnostic characteristics. Our results suggested the pooling of several peptides to reach a high level of sensitivity required for reliable immunodiagnosis.

## Introduction

For serodiagnosis of human helminthic infections, many currently used tests rely on native antigens, either extracted from whole worms (somatic antigens) maintained in laboratory animals, or cultivated *in vitro* to obtain excretory/secretory products (metabolic antigens). These natural antigens are limited in availability and suffer from batch-to-batch variation. A number of *Echinococcus* proteins have been recombinantly produced and tested for use in serodiagnosis [1]–[6] but to our knowledge, only recombinant EmII/3-10 [7], [8] and its related sequence Em18 [9] are successfully applied in commercial test kits. Recombinantly expressed antigens used in diagnostic tests require a high degree of purification to avoid cross-reactivity due to contaminants from the expression system. Unspecific binding and cross-reactivity are major problems with both, extracts of whole worms [10], [11] and recombinant proteins [12]. For improving diagnostic test performance, it is desirable to identify highly specific and highly reactive epitopes from the proteome of the pathogen in question and synthetically produce the corresponding peptide antigens. Synthetic peptides are advantageous for diagnostic applications since they are well defined, easily produced in large amounts, highly pure and often cost-saving if compared to the production of natural antigen in animal models or *in vitro* culture. Applications of peptides in immunodiagnosis of different parasitic diseases were given by Noya et al. [13].

The availability of an increasing number of pathogen genomes is boosting basic research as well as applied science. In the field of parasitological diagnostics, sequencing of parasites genomes also creates new opportunities. Annotated genomes and proteomes are available for some of the medically important protozoan pathogens, such as *Plasmodium* species and some Kinetoplastida species. Following the genome of the parasitic nematode *Brugia malayi* [14], the genome of *Schistosoma mansoni*, a blood vessel dwelling trematode, has recently been released [15] as well as a draft genomic sequence for *Schistosoma japonicum* [16] and more genome data from various helminth species are to be expected in the near future (see for example http://www.sanger.ac.uk/Projects/Helminths/ or http://www.nematode.net/). Currently, proteins predicted from ESTs, whole genomes or contigs are available for data mining already from a considerable number of helminth species. Using the sequence data available in the public domain, we have designed and tested a pathway for identifying novel antigens for serodiagnostic test development.

Our selection procedure for peptide antigens relied on the application of bioinformatic filters to collections of protein sequences, including conceptually translated nucleotide sequence data. The selection criteria aimed at proteins located to the host-parasite interface. Such proteins are potentially seen by the host immune system and may elicit an immune response and thus represent good candidates of diagnostic antigens. Our *in silico* analysis therefore prioritized proteins containing a sorting signal directing the protein to the extracellular space (PSORT II [17]), transmembrane domains (TMHMM II [18]), or a C-terminal signature sequence for addition of glycosyl phosphatidylinositol (GPI) anchor (GPI-som [19]). These prediction algorithms require full length amino acid sequences with complete N- and C-termini, thus excluding the use of most EST libraries. The majority of proteins generally display well defined three dimensional structures, which is mostly globular. Our selection procedure preferred sequences in which the chemically synthesized peptide also adopts a natural conformation *in vitro*. There are two structural motifs meeting this claim. Firstly, the intrinsically unstructured regions (IUR) that lack a well defined three dimensional structure displaying an extended conformation that can be identified for example by IUPred [20]. Secondly the alpha-helical coiled-coil motif predicted for example by Paircoil2 [21]. Alpha-helical coiled-coils are believed to readily fold into a stable structure in aqueous solution [22]. A microarray platform served for testing the diagnostic potential of the peptides selected *in silico*. Using a high-throughput microarray format brings together the advantages of low reagent consumption and rapid multiplexed analysis. Particularly in diagnostics, the possibility of testing the reactivity of a given serum sample with multiple antigens simultaneously harbors great benefits. Promising candidates identified on microarray were further explored and validated for use in ELISA.

We have carried out the proof-of-principle for bioinformatic selection of suitable long synthetic peptides (LSPs) for the tapeworms *Echinococcus multilocularis* and *Echinococcus granulosus*, both of major medical importance causing alveolar (AE) and cystic echinococcosis (CE), respectively. CE and particularly AE, due to its infiltrative and/or space-occupying growth, are severe diseases with high fatality rate and poor prognosis if managed incorrectly [23]. The importance of alveolar echinococcosis is not represented by the number of reported cases but rather by the severity of the disease in the individual patient [24]. Standard diagnostic tests for human echinococcosis, AE as well as CE, imply imaging techniques such as ultrasonography, x-ray and computer tomography, as well as serological tests based on enzyme-linked immunosorbent assay (ELISA) and immunoblots [25]. Antigens used in these serodiagnostic tests were developed from crude extracts to purified fractions to recombinant antigens and for *E. multilocularis* to vesicular fluid originating from *in vitro* cultivated metacestodes [26]. Despite substantial improvements have been achieved, the main screening test still relies on the availability and quality of native antigens, i.e. hydatid fluid of *Echinococcus granulosus* cysts collected from naturally infected intermediate hosts at the slaughterhouse. One study reported that source and quality or fertility of cysts are critical for test outcome. This called for standardization of antigens and test methods [10]. To produce a robust and reproducible test for the routine diagnostic laboratory, we have evaluated the performance of chemically synthesized peptides in comparison to natural (EgHF, EM2) [26], [27] and recombinant antigen (EmII/3-10) [7].

## Materials and Methods

### Ethics statement

Ethical clearance for retrospective use of anonymized patient sera for test development and quality control was obtained from the ethical committee (Ethikkommission beider Basel).

### Human serum samples

Sera of healthy blood donors living in Switzerland were used to define a cut-off for distinguishing between positive and negative test results. In ELISA, 50 blood donor sera were used. In microarray, a single serum and 2 pools made of 5 sera each were used. For testing diagnostic peptide reactivity in ELISA, 44 sera from *E. multilocularis-* and 35 sera from *E. granulosus*-infected patients from Central Europe were used. All echinococcosis patients had active hepatic lesions of either CE1 or CE2 type (WHO-IWGE standardized classification) and all sera were sampled prior to any therapeutic intervention, i.e. before surgery and/or chemotherapy. Diagnoses were confirmed serologically as described by Müller et al. [26], in complementation to the clinical diagnosis based on imaging procedures and, if available, retrospective histopathological investigations. For testing cross-reactivity of the peptides, sera from patients with following infections were used (concomitant echinococcosis was ruled out by clinical and serological criteria): 2 trichinellosis, 2 trichuriasis, 10 toxocariasis, 8 ascariasis, 1 anisakiasis, 2 hookworm infection, 8 strongyloidiasis, 10 filariasis (*Loa loa*, *Mansonella perstans*, *Onchocerca volvulus*), 10 fascioliasis, 1 paragonimiasis, 11 schistosomiasis (*Schistosoma mansoni*, S. *haematobium*, S. *mekongi*), 12 neurocysticercosis (*Taenia solium*), 1 taeniasis (*Taenia saginata*), 1 diphyllobothriasis and 26 amoebic liver abscess (*Entamoeba histolytica*).

### Sequence selection and peptide design

Protein sequences of *Echinococcus multilocularis* and *E. granulosus* were retrieved from NCBI Entrez Protein Database on October 16, 2007. NCBI Entrez Protein Database are compiled from a variety of sources with daily updates, including SwissProt, PIR, PRF, PDB, and translations from annotated coding regions in GenBank and RefSeq. To identify putative membrane or extracellular proteins, the protein sequences were analyzed using TMHMM II [18] for prediction of transmembrane domains, GPI-SOM [19] for prediction of glycosylphosphatidylinisotol (GPI) anchor signals, and PSORT II [17] for prediction of protein subcellular localization. All protein sequences were further screened for stretches of alpha-helical coiled-coils using Paircoil2 [21] and intrinsically unstructured regions using IUPred [20]. Both of these structures are believed to adopt native conformation in aqueous solution and therefore constitute major selection criteria. Due to the limited number of *Echinococcus* sequences in NCBI Entrez Protein Database and few predicted surface proteins containing stretches of alpha-helical CC and/or IURs, the analysis was extended to all proteins with alpha-helical coiled-coil and IUR predictions, irrespective of their predicted locations. This included known proteins previously tested in immunodiagnosis. To narrow down protein regions for selection of 30mer peptides, protein sequences were subjected to prediction of coiled-coil stability by STABLECOIL (http://biomol.uchsc.edu/cores/biophysics/stablecoil). In case of IURs antigenicity predictions were performed using BepiPred [28]. Thus detected regions of high predicted CC stability or antigenicity were favored. To increase solubility, peptides were selected to start and end with a hydrophilic or neutral amino acid and to harbour on average one charged amino acid, either positive or negative, per five residues. In addition, the guidelines suggested by the manufacturer were followed (http://www.altabioscience.bham.ac.uk/pdfs/Intro_to_series_SYNTHETIC_PEPTIDES.pdf). From 11 sequences listed in table S1, 45 peptides were selected according to the criteria mentioned above.

Peptides were produced by Fmoc solid phase synthesis (Alta Bioscience, University of Birmingham, UK). The length of the peptides was limited by the EpiScan synthesis procedure (Alta Bioscience, University of Birmingham, UK) to a maximum of 30 amino acids. Thus the length of the peptides used for spotting onto microarrays ranged between 24 and 30 amino acids with an additional aminohexanoid acid (AHX) spacer and a biotin at the N-terminus. Biotin was required to bind peptides to a streptavidin-coated solid phase, i.e. microscope glass slide for microarrays (Alta Bioscience, University of Birmingham, UK) or to 96 well plates for ELISA. In order to remove electric charges from the free C-terminus, the synthetic peptides were modified by carboxy-terminal amidation. Thus, both N-terminal and C-terminal peptide ends were uncharged, mimicking natural segments of internal protein sequences.

For defining optimal peptide length, we designed extended variants of the 8 most reactive candidates from the microarray. The length of extended peptides ranged between 40 and 47 residues (table 1). Modifications at the N- and C-terminus were identical to those of the microarray peptides. The extension of peptides was chosen to increase alpha-helical coiled-coil stability or to combine epitopes from two single peptides. To target these improved peptides to *E. multilocularis* diagnosis, we chose the *E. multilocularis* sequences for designing longer peptides. Sequences derived from antigen B8/1 (accession number BAC77657) and antigen B8/2 (accession number BAD89809). To our knowledge, these were the first synthetic peptides from any *E. multilocularis* antigen B sequence that were serologically evaluated. Alignments of homologous antigen B8/1- and antigen B8/2-sequences of the two *Echinococcus* species are shown in figures S1 and S2, respectively. Antigen B8/1-sequences of *E. granulosus* and *E. multilocularis* showed 86% identity and antigen B8/2-sequences 93% identity. Peptide longD8-9 coincided with the region of a previously published *E. granulosus* epitope EVKYFFER [29], but differed from another published synthetic peptide p176 [30]: p176 spans the N-terminal region of antigen B8/1 (amino acids 17–54), while longD8-9 covered the central part of the protein (amino acids 33–74). Within the overlap of 26 amino acids, three amino acids differed.

**Table 1:** Original and extended peptides of the 8 most reactive candidates from microarray.

Protein	Accession number	Microarray peptide	Length	Accession number	Extended peptide	Length	Peptide sequence
EmII/3	AAA50580	D1	29	AAA50580	longD1	43	EQKLRELRAQMVEKESDLADMKNKASAYESKIAELEMLLQQER
EmII/3	AAA50580	D12	25	AAA50580	longD12	40	DEVQREVEAQKVAMAKKEAEKAQAEAELRRMREKHDAKHK
AgB8/1	AAD38373	D8, D9	29	BAC77657	longD8-9	42	KMLGEMKYFFERDPLGQKLVDLLKELEEVFQMLRKKLRTALK
AgB8/2	AAC47169	D11	27	BAD89809	longD11	45	DPLGQRLVALGNDLTAICQKLQLKIREVLKKYVKNLVEEKDDDSK
EM13	Q07840	A9	29	Q07840	longA9	45	QVQNAKNEPFGTPEQLRKIEDKLRKGIMEEEKTRKAYEEALSSLS
PSCCP*	CAD44854	B6	25	CAD44854	longB6	47	ETIQSLCEHNAALQQKLDEANQSVTEVSVQMKVMQQHLHTARVAIQS
EG19	ABI24154	D3	27	ABI24154	longD3	42	EAEAKCLRRPHQRVVKEGEVSKGDEVDGEDRDDECVGGDEGR

*PSCCP: protoscolex-specific coiled-coil protein.

### Microarray

A microarray platform was used for screening *in silico* selected peptides for reactivity with patients' sera. The low-density peptide microarray (Alta Bioscience, University of Birmingham, UK) featured two blocks, each containing 45 *Echinococcus* peptide spots, a spotting control allowing positioning of grid for analysis (unrelated, TAMRA-labeled peptide), a control for serological detection (human IgG) and a blank (spotting solution). All microarray work was done at room temperature. Microarray slides were blocked for one hour in assay buffer (1×PBS pH 7.4, 0.05% Tween20, 3% milk powder). Individual human sera or serum pools were diluted 1∶50 in assay buffer and incubated for two hours in a moist chamber. Slides were washed for 3×5 minutes in assay buffer and incubated for one hour with a 1∶100 dilution of Cy5-labeled goat anti-human IgG (H+L) secondary antibody (Jackson ImmunoResearch Laboratories product number 109-175-088). Slides were washed for 3×5 minutes in assay buffer and quickly rinsed with deionized water before drying with compressed air. Slides were scanned at 532 and 635 nm using a GenePix 4100A microarray scanner (Bucher Biotec AG, Basel, Switzerland). Cy5 and TAMRA images and local background corrected fluorescence intensities (FI) were acquired using the Axon GenePix Pro 6.0 software. Further analysis was done in Microsoft Excel. Duplicate FI-values were averaged. Peptides were determined reactive if FI>1009. This cut-off was determined by testing single or pooled blood donor samples (pools made of 5 sera each), calculating average FI intensities plus 4 times the standard deviation. We chose 4 standard deviations rather than 3 because the majority of duplicate values differed in more than 20% from each other. This variation can be explained by unequal spot morphology. Peptides from reactive spots were forwarded to evaluation of test sensitivity and specificity performed by ELISA, the current standard format of routine serodiagnostics.

### ELISA

96-well plates (NUNC Immobilizer Streptavidin) were pre-washed three times using an ELISA plate washer (deionized H_2_O, 0.05% Tween20) and coated overnight at 4°C with 100 µl/well of synthetic peptide diluted to 2 µg ml^−1^ in PBS pH 7.4. All following steps were carried out at room temperature. After washing, the plates were blocked for one hour with 150 µl/well of assay buffer (3% milk in PBS pH 7.4, 0.05% Tween20, 0.5 mM biotin). The plates were washed and incubated for one hour with human sera diluted 1∶200 in assay buffer (100 µl/well). Alkaline-phosphatase-conjugated goat anti-human IgG antibodies (Sigma product number A 3187) 1∶1000 in assay buffer were used as secondary antibodies. After washing, 100 µl of conjugate dilution was added to each well and incubated for one hour, followed by a final wash. Wells were then incubated for 15 minutes with 100 µl of *p*-nitrophenyl phosphate (Sigma product number N 4645) at a concentration of 1 mg ml^−1^ in substrate buffer (13.2 mM Na_2_CO_3_, 35 mM NaHCO_3_, 1mM MgCl_2_×6H_2_O, pH 9.6). Absorbance values (A_405nm_) were measured at 405 nm in a Tecan Sunrise microplate absorbance reader. All serum samples were tested in duplicates. The two values were averaged and blank-corrected. For distinguishing between positive and negative ELISA test results, 50 sera from healthy blood donors living in Switzerland were tested. The cut-off value was determined by the mean of the blood donor samples plus two standard deviations. Sensitivity and specificity of the single peptide candidates were calculated using test results from confirmed echinococcosis patients as true positives (TP). Blood donors and individuals infected by other helminths were taken as true negatives (TN). The working characteristics of the peptide antigens were explored by receiver-operating characteristics (ROC) plot analysis (Analyse-It, version 2.21).

To test the impact of pooling peptide candidates, LSP longD12, longD1 and longD8-9 were mixed in equal parts (mixW) and applied at the same conditions as described above. The cut-off was determined by the mean A_405nm_ of fifty blood donor samples plus 2 standard deviations. Sensitivity and cross-reactive behavior was tested with 30 echinococcosis sera and 15 sera from various helminth and amoeba infections that had previously shown cross-reactivity with the single peptides.

## Results

### Peptide selection

240 entries of *E. multilocularis* and 940 entries of *E. granulosus* proteins were retrieved from NCBI Entrez Protein Database including redundancies. Combining the outputs of PSORT II, TMHMM II, GPI-som, IUPred and Paircoil2, led to the selection of 11 protein candidates (table S1). According to the information available in GenBank, these proteins were isolated from metacestode or protoscolex tissue, with the possible exception of HSP70, for which no information on developmental stage was provided.

Six proteins had been reported previously to react with antibodies from patients' sera: EmII/3 [7], EM13 [6], P-29 [31], GRP [32], EG19 [33], AgB8/1 and AgB8/2 [1]. These earlier findings supported the inclusion of these antigens in our analysis. In total, 45 peptide sequences from 11 proteins were chosen to be chemically synthesized and spotted onto microarray. 31 peptides derived from *E. multilocularis* and 14 from *E. granulosus* (table S1).

### Screening by peptide microarray

*In silico* selected peptides were screened for diagnostic potential on a microarray platform (Alta Bioscience, University of Birmingham, UK). From a total of 45 peptides contained in the microarray, 17 showed reactivity with pooled or single sera from echinococcosis patients (8/31 peptides from *E. multilocularis*, 9/14 peptides from *E. granulosus*). The peptides that reacted above the cut-off determined from blood donor sera (FI>1009) are listed in table 2. These 17 peptides were considered to have diagnostic potential and were therefore subjected to further testing of specificity and sensitivity. The predicted structure of the reactive peptide candidates was mostly alpha-helical coiled-coil (7 peptides) and alpha-helical (7 peptides). 3 peptides were predicted to be intrinsically unstructured.

**Table 2:** Peptides with diagnostic potential identified on microarray.

	*E.m.*	*E.m.*	*E.m.*	*E.m.*	*E.m.*	*E.m.*	*E.m.*	*E.m.*	*E.g.*	*E.g.*	*E.g.*	*E.g.*	*E.g.*	*E.g.*	*E.g.*	*E.g.*	*E.g.*
Protein	a)	a)	a)	b)	b)	b)	c)	c)	d)	d)	e)	e)	f)	f)	f)	g)	g)
Peptide name	A8	A9	A10	A4	A5	D12	B6	B9	C10	D2	D3	D5	D7	D8	D9	D10	D11
Structure	helical	helical	IUR	CC	CC	CC	CC	CC	helical	helical	IUR	IUR	helical	helical	helical	helical	CC
Em-pool-1	1	1	0	0	0	1	1	0	0	1	0	1	1	1	0	0	0
Em-pool-2	0	0	0	0	0	0	0	0	0	0	0	0	0	0	0	0	0
Em-pool-3	0	0	0	0	1	0	0	0	0	0	0	0	1	1	0	0	0
Em-pool-4*	1	1	1	0	0	1	1	1	0	0	1	0	0	1	0	0	1
Eg-pool-1	0	0	0	0	0	0	0	0	0	0	0	0	1	1	0	1	1
Em-3	0	1	0	0	0	0	0	0	0	0	0	0	0	0	0	0	0
Em-11	0	1	0	0	0	0	0	0	0	0	0	0	0	1	0	0	0
Em-12	0	0	0	0	0	0	0	0	0	0	1	0	0	0	0	0	0
Em-23	0	0	0	0	0	0	0	0	0	0	1	0	0	1	0	0	1
Em-24	0	0	0	0	0	1	0	0	0	0	0	0	0	0	0	0	0
Em-2	0	0	0	0	0	0	0	0	0	0	0	0	0	0	0	0	0
Em-15	0	1	0	0	0	1	0	0	0	0	0	0	1	0	1	0	1
Em-18	0	0	0	0	0	0	0	0	1	0	0	0	1	0	0	0	0
Em-34	0	0	0	0	0	0	0	0	0	0	0	0	0	0	0	0	0
Em-35	0	0	0	1	0	0	0	0	0	0	0	0	1	0	0	0	0

*Data incorporates merged results from repeated assays.

a) EM13 (accession Q07840); b) EmII/3 (accession AAA50580); c) Protoscolex specific coiled-coil protein (accession CAD44854); d) HSP70 (accession Q24789); e) EG19 (accession ABI24154); f) Antigen B8/1 (accession AAD38373); g) Antigen B8/2 (accession AAC47169).

1 = positive.

0 = neative.

Em = AE serum.

Eg = CE serum.

### Assessing peptide performance in ELISA

**Figure 1:**
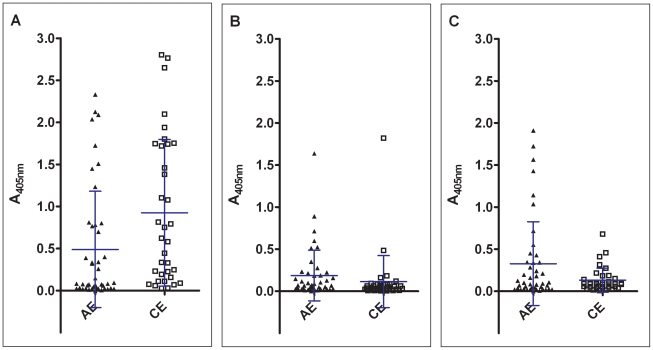
No clear discrimination of A_405nm_ values measured from alveolar echinococcosis (AE) and cystic echinococcosis (CE) sera. Following peptides served as antigens: 1A) longD8-9, cut-off 0.165. 1B) longD1, cut-off 0.200. 1C) longD12, cut-off 0.357. Bars represent mean and standard deviation. ▴ Alveolar echinococcosis (AE); □ Cystic echinococcosis (CE).

The 17 peptides reactive on microarray were carried forward for evaluating their application in ELISA-based serodiagnosis, the current routine technique. Test sera from AE and CE patients and sera from patients with infections other than echinococcosis were used. Sensitivity, cross-reactivity and impact of peptide length were evaluated (data not shown). With respect to sensitivity, long peptides (more than 30 residues) were superior to shorter ones. 3 out of 17 peptides were selected for extensive validation: longD12 (deriving from *E. multilocularis* EmII/3-10), longD1 (EmII/3-10) and longD8-9 (*E. multilocularis* antigen B8/1). All 3 peptides did not clearly differentiate between AE and CE infections as can be seen by overlapping standard deviations of A_405nm_ values obtained from AE and CE sera (figure 1). Peptides longD1 and longD12 derived from EmII/3-10, a well established antigen specific for the diagnosis of *E. multilocularis* infections [8]. In our hands, longD12 reacted with both, AE and CE sera (table 3: AE 10/44; CE 3/35). This contrasts with previous observations of a specific reactivity of EmII/3-10 with AE sera. The reactivity of peptide longD1 was similar to longD12 (AE: 11/44; CE: 2/35). Peptide longD8-9 (*E. multilocularis* antigen B8/1) reacted with more CE than AE sera (AE: 19/44; CE: 26/35) (table 3).

**Table 3:** Numbers of sera tested positive with peptides longD12, longD1 and longD8-9.

		Peptide	longD8-9	longD1	longD12	Cumulative result
		Cut-off A_405nm_	0.165	0.200	0.357	
		Total tested	Positive	Positive	Positive	Positive
Healthy	Blood donors	50	4	4	4	11
Cestoda	AE	44	19	11	10	28
	CE	35	26	2	3	27
	Taeniasis	1	0	0	0	0
	Cysticercosis	12	1	0	0	1
	Diphyllobotriasis	1	0	0	0	0
Nematoda	Trichinellosis	2	0	0	0	0
	Trichuriasis	2	0	0	0	0
	Anisakiasis	1	0	0	0	0
	Hookworm	1	0	0	0	0
	Strongyloidiasis	8	1	2	1	2
	Toxocariasis	10	0	1	1	2
	Filariasis	10	0	1	0	1
	Ascariasis	8	0	0	0	0
Trematoda	Paragonimiasis	1	0	0	0	0
	Fascioliasis	10	0	1	0	1
	Schistosomiasis	10	1	3	2	5
Protozoa	Amoebiasis	26	3	4	3	8

**Figure 2:**
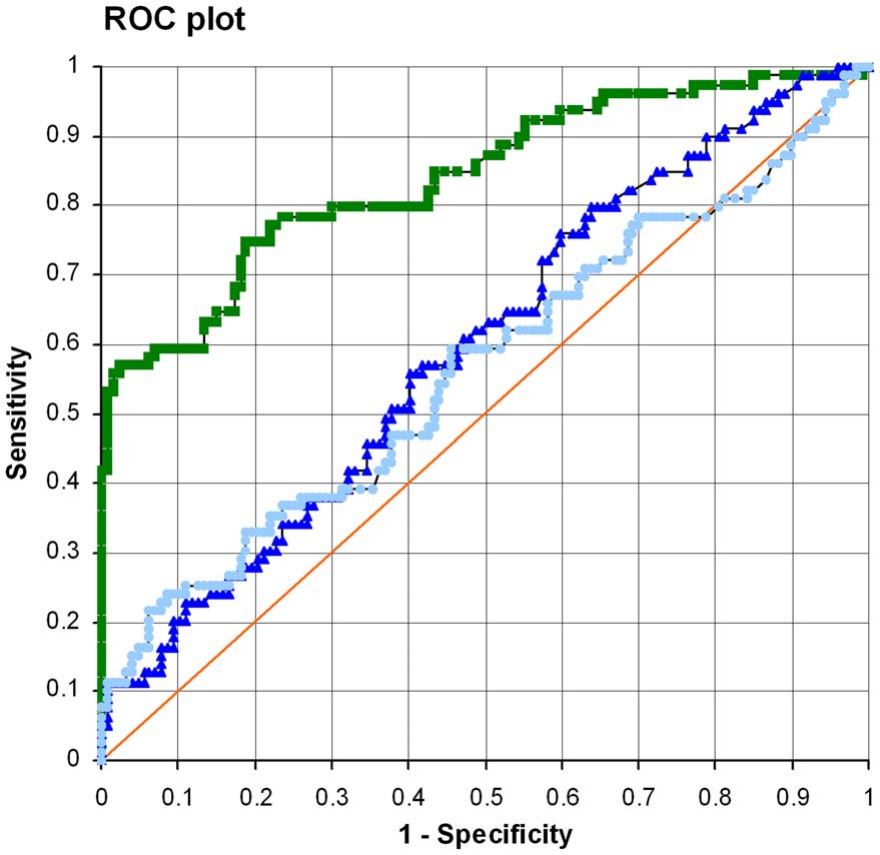
ROC plot illustrating sensitivity and specificity of LSP longD12, longD1, longD8-9. Diagonal line (orange): No discrimination; Square (green): longD8-9; Triangle (dark blue): longD1; Circle (light blue): longD12.

The cut-off values determined from 50 blood donors were similar for longD8-9 and longD1 with 0.165 and 0.200, respectively. The cut-off for longD12 was higher with an OD of 0.357. Compared to 90% sensitivity of a commercial *E. granulosus* hydatid fluid ELISA used in routine testing [34], the sensitivity of each single peptide alone was rather low, candidate longD8-9 being the most sensitive with 57%. LongD1 and longD12 both reached 16%. The specificities of longD8-9, longD1 and longD12 were 94%, 91% and 94% respectively, thus performing in the range required for a routine diagnostic assay. There were minor cross-reactivities observed with sera from helminth infections other than echinococcosis (table 3). Peptide longD8-9 was positive with 1/12 cysticercosis, 1/8 strongyloidiasis and 1/10 schistosomiasis sera. Peptide longD1 was seropositive with 2/8 strongyloidiasis, 1/10 toxocariasis, 1/10 filariasis, 1/10 fascioliasis and 3/10 schistosomiasis sera. Peptide longD12 was positive with 1/8 strongyloidiasis, 1/10 toxocariasis, 2/10 schistosomiasis sera. Cross-reactions with *Taenia*, *Strongyloides* and filaria are also seen in conventional diagnostic assays. We further tested the specificity with sera from patients suffering from liver abscess due to *Entamoeba histolytica* infection. LongD12 was positive with 3/26, longD1 with 4/26 and longD8-9 with 3/26 amebiasis sera (table 3). To compare the diagnostic performance of each peptide, a receiver-operating characteristics (ROC) plot was generated (figure 2). Candidate longD8-9 emerged as clear favorite, although on its own not meeting the sensitivity required for antigens applied in routine diagnostics.

The use of 30–50mer peptides for serology impairs sensitivity compared to the full length antigen, because peptides likely harbor less epitopes. We calculated the cumulative sensitivity of the 3 single peptide ELISAs and measured the experimental combination of the 3 peptides in one antigen mix (mixW). The cut-off of mixW was 0.352, which is in the same range as the cut-off of one of its components, namely longD12. By cumulating the individual results of the 3 assays, the theoretical sensitivity indeed increased from 57% to 70%, but specificity decreased to 82% (table 4). Compared to cumulative positivity, pooling the 3 peptides in one well (mixW) led to a loss of positive test results. In return, the specificity increased due to a loss of cross-reactions. Positive test results obtained by single peptide ELISAs compared to pooled peptide ELISA are summarized in table 5.

**Table 4:** Sensitivity and specificity of peptides longD12, longD1 and longD8-9.

Peptide	total AE+CE	TP	FN	TN	FP	Sensitivity	Specificity
longD8-9	79	45	34	120	7	57%	94%
longD1	79	13	66	115	12	16%	91%
longD12	79	13	66	127	8	16%	94%
cummulative	79	55	24	104	23	70%	82%

TP = true positive, FN = false negative, TN = true negative, FP = false positive.

**Table 5:** Comparison of positivity achieved by single peptides, cumulative results of single peptides and peptide pool mixW.

	longD8-9	longD1	longD12	cumulative result	mixW
Em-1	1	1	0	1	1
Em-5	1	0	0	1	0
Em-9	1	1	0	1	1
Em-14	1	1	0	1	1
Em-27	1	0	0	1	1
Em-31	0	0	1	1	0
Em-38	0	1	0	1	1
Em-39	1	0	1	1	1
Em-40	1	0	0	1	0
Em-41	1	1	0	1	1
Em-42	0	1	0	1	1
Em-44	1	0	0	1	1
Em-45	0	1	1	1	1
Em-47	0	0	1	1	1
Em-53	1	0	0	1	1
Eg-1	1	0	1	1	1
Eg-2	1	0	0	1	0
Eg-3	1	0	0	1	0
Eg-11	1	0	0	1	1
Eg-12	0	0	0	0	0
Eg-13	1	0	0	1	0
Eg-14	1	1	0	1	1
Eg-22	1	0	0	1	1
Eg-24	1	0	0	1	0
Eg-25	1	0	1	1	1
Eg-28	1	0	0	1	1
Eg-29	1	0	0	1	1
Eg-31	1	0	0	1	1
Eg-32	1	0	0	1	1
Eg-34	0	1	0	1	0
N-7	1	1	1	1	1
N-24	0	0	1	1	1
N-25	0	1	0	1	0
N-35	0	0	0	0	0
T-15	0	0	0	0	0
T-16	0	0	1	1	1
T-17	1	1	0	1	0
T-20	0	1	0	1	0
C-10	1	0	0	1	0
C-12	0	0	0	0	0
C-14	0	0	0	0	0
A-4	0	1	1	1	1
A-23	1	0	0	1	0
A-24	1	0	0	1	1
A-26	0	0	1	1	1

1 = positive.

0 = negative.

Em = *E. multilocularis* infection.

Eg = *E. granulosus* infection.

N = nematoda infection.

C = cestoda infection.

T = trematoda infection.

A = amoeba infection.

## Discussion

The use of synthetic peptides as substitutes of native antigen in immunoassays has been highly recommended for the diagnosis of infectious diseases by different authors [13], [30], [35]. Synthetic peptides are applied successfully in diagnosis of viral and bacterial infections [36]–[39]. Compared to a metazoan parasite, viruses and bacteria in general have smaller genomes and accomplish less complex post translational protein modifications, which might imply more rapid success in identification of immunodominant peptides. It even allows for analysis on whole proteome level [40]. Here, we investigated the use of synthetic peptides of 30–50 amino acids in length for the use in immunodiagnosis of human hydatid disease. We provided proof of principle for the selection of synthetic peptides by bioinformatic means and for screening on a microarray platform for diagnostic reactivity. 17 out of 45 peptides on the microarray proofed reactive with sera from echinococcosis patients.

The peptide performing best in our investigation was longD8-9, a 42mer peptide deriving from *E. multilocularis* antigen B8/1. It reached a sensitivity of 57% and a specificity of 94%. The reactivity of LSP longD8-9 with sera from AE as well as CE patients suggests its use for both, patient screening as well as for follow-up examinations of treated patients. For the latter purpose, it will be necessary to determine the proportion of patients seroconverting during treatment. The applicability of our peptide antigen for monitoring treatment follow-up samples is ongoing work in our laboratory.

Our results confirm previous reports [38], [41] that a test based on a single peptide needs to be complemented by additional peptides to reach a sensitivity comparable to the antigen extract. To compensate for limited sensitivity of individual peptides, we performed ELISAs with combinations of synthetic peptides applied as mixtures for coating. We compared these results to the “cumulative sensitivity”, a theoretical value obtained by summing up the positive results from individual peptide ELISAs for each serum tested. The cumulative result amounted to a theoretical sensitivity of 70%. We did not detect this high sensitivity. In contrast, pooling of 3 peptides in one well led to a loss of positive signals otherwise obtained with individually tested peptides. 8/29 echinococcosis samples were lost through pooling. One reason was suggested by an increased cut-off for pooled peptides. In some sera similar ODs were observed in individual test and in peptides mixtures, but due to increased cut-off for pooled peptides, these sera turned negative. The loss of sensitivity obtained with pooled peptides in mixW could also be due to partial occupation of streptavidin binding sites with less reactive antigen. The same reasons may account for increased specificity. Combination of peptides in one well led to a loss of unwanted cross-reactivity with 5/11 previously false positive signals disappearing. Thus in our study, pooling peptides increased specificity, but lowered sensitivity. In future work peptide mixes might be optimized by combining peptides with equally good diagnostic sensitivity and specificity. In particular, the sensitivity of mixes is likely increased by continuous search for new peptides with improved diagnostic operating characteristics as substitutes for the least reactive candidates. The behaviour of peptide mixtures applied as antigens in ELISA needs further investigation.

To select peptides with broad recognition by human echinococcosis patients, our bioinformatic selection aimed at exported or parasite surface exposed antigens. Such proteins are likely to elicit an immune response. A major drawback was the scarce number of full-length *Echinococcus* spp. protein sequences deposited in public databases. Thus it might be worthwhile for further studies to adapt the bioinformatic selection criteria to the use of partial EST sequences. Our data points out the need for screening more peptide candidates. Further investigations on whole genome level are likely to provide additional candidates with sufficient sensitivity and specificity, which can eventually be combined to a synthetic antigen pool. Single or pooled LSPs are compatible with high throughput platform technologies such as luminex or biacore technology that could replace ELISA in the future.

Additionally to surface or extracellular localization, one criterion during bioinformatic selection was prediction of secondary structures. Alpha-helical coiled-coils and intrinsically unstructured proteins are believed to adopt native conformation in aqueous solution and might therefore be well suited for immunoassays. It is widely accepted that the majority of epitopes are of the conformational type [42]. Our results showed that for diagnostic purposes the predicted alpha-helical and alpha-helical coiled-coil peptides were superior to peptides deriving from intrinsically unstructured regions. Among the serum-reactive peptides identified by microarray screening, 14 peptides were of predicted alpha-helical organization, while only 3 peptides derived from intrinsically unstructured regions. Furthermore, during our extensive testing of candidates, all IUR peptides were dismissed due to lack of sensitivity.

In addition to the secondary and tertiary structure of a peptide, a further determinant for recognition is the immobilization of peptides to the solid phase used in an immunodiagnostic test. Antibody capture by peptides crucially depends on the accessibility of key residues and therefore the introduction of a spacer molecule to spatially separate the peptide from its carrier protein is vitally important [43]. The peptides in our study were synthesized with an N-terminal AHX-spacer coupled to biotin. The biotin was used to immobilize the peptide antigen to a streptavidin-coated solid phase, i.e. microscope glass slides and 96-well ELISA plates. Coating the biotinylated peptides to non-streptavidin surfaces such as Immulon 2HB (Thermo Scientific) or Poly Sorp (NUNC) ELISA plates, led in those samples tested to discrepancies of duplicate values and mostly reduced A_405nm_ values, in a non-linear manner (data not shown). Immobilization of peptides by direct adsorbance to the polystyrene surface might lead to sterical blockage of key residues necessary for interaction with antibodies and thus decrease the A_405nm_ values. Similarly, HIV-1 peptides showed in ELISA increased test sensitivity and specificity when immobilized via biotin-streptavidin [44]. Surfaces functionalized with streptavidin guarantee a directional, highly dense and reproducible coating of biotinylated peptide antigens. It has been shown that the direction of peptide immobilization is of secondary importance as long as peptides deriving from internal protein sequences are investigated [43]. Interactions of internal sequences do not depend on free N- or C-terminus and therefore attachment of spacer and carrier molecules can be achieved successfully at both termini of the peptide.

Our approach represents an alternative to peptide selection by phage display [45], [46]. The bioinformatic selection avoids construction and handling of phage display libraries and panning procedures. The most important limitation to successful panning consists in the lack of selective antibodies. Antibody purification from human sera requires pure and specific antigen, which generally is not yet available. Our bioinformatic approach to peptide selection reduces complex lab work and is compatible with screening on peptide microarray. In our hands, this platform proved highly suitable for investigation of antigen-antibody interaction.

## Supporting Information

Figure S1 Alignment of *E. granulosus* and *E. multilocularis* antigenB8/1 sequences with location of peptide D8, D9 and longD8-9.
(0.42 MB TIF)

Figure S2 Alignment of *E. granulosus* and *E. multilocularis* antigenB8/2 sequences with location of D11 and longD11.
(0.44 MB TIF)

Table S1 Final set of peptides spotted onto microarray.
(0.12 MB DOC)

## References

[1] Rott MB, Fernandez V, Farias S, Ceni J, Ferreira HB (2000). Comparative analysis of two different subunits of antigen B from *Echinococcus granulosus*: gene sequences, expression in *Escherichia coli* and serological evaluation.. Acta Trop.

[2] Mamuti W, Yamasaki H, Sako Y, Nakao M, Xiao N (2004). Molecular cloning, expression, and serological evaluation of an, 8-kilodalton subunit of antigen B from *Echinococcus multilocularis*.. J Clin Microbiol.

[3] Lorenzo C, Last JA, Gonzalez-Sapienza GG (2005). The immunogenicity of *Echinococcus granulosus* antigen 5 is determined by its post-translational modifications.. Parasitology.

[4] Li J, Zhang WB, Wilson M, Ito A, McManus DP (2003). A Novel Recombinant Antigen for Immunodiagnosis of Human Cystic Echinococcosis.. Jour Infect Dis.

[5] Hernández-González A, Muro A, Barrera I, Ramos G, Orduña A (2008). Usefulness of four different *Echinococcus granulosus* recombinant antigens for serodiagnosis of unilocular hydatid disease (UHD) and postsurgical follow-up of patients treated for UHD.. Clin Vaccine Immunol.

[6] Frosch PM, Geier C, Kaup FJ, Müller A, Frosch M (1993). Molecular cloning of an echinococcal microtrichal antigen immunoreactive in *Echinococcus multilocularis* disease.. Mol Biochem Parasitol.

[7] Vogel M, Gottstein B, Müller N, Seebeck T (1988). Production of a recombinant antigen of *Echinococcus multilocularis* with high immunodiagnostic sensitivity and specificity.. Mol Biochem Parasitol.

[8] Müller N, Gottstein B, Vogel M, Flury K, Seebeck T (1989). Application of a recombinant *Echinococcus multilocularis* antigen in an enzyme-linked immunosorbent assay for immunodiagnosis of human alveolar echinococcosis.. Mol Biochem Parasitol.

[9] Sako Y, Nakao M, Nakaya K, Yamasaki H, Gottstein B (2002). Alveolar echinococcosis: Characterization of diagnostic antigen Em18 and serological evaluation of recombinant Em18.. J Clin Microbiol.

[10] Poretti D, Felleisen E, Grimm F, Pfister M, Teuscher F (1999). Differential immunodiagnosis between cystic hydatid disease and other cross-reactive pathologies.. Am J Trop Med Hyg.

[11] Yamano K, Goto A, Nakamura-Uchiyama F, Nawa Y, Hada N (2009). Gal beta 1-6Gal, antigenic epitope which accounts for serological cross-reaction in diagnosis of *Echinococcus multilocularis* infection.. Parasite Immunol.

[12] Müller N, Felleisen R (1995). Bacterial expression systems as tools for the production of immunodiagnostic parasite antigens.. Parasitol Today.

[13] Noya O, Patarroyo ME, Guzman F, de Noya BA (2003). Immunodiagnosis of parasitic diseases with synthetic peptides.. Curr Protein Pept Sci.

[14] Ghedin E, Wang S, Foster JM, Slatko BE (2004). First sequenced genome of a parasitic nematode.. Trends Parasitol.

[15] Berriman M, Haas BJ, LoVerde PT, Wilson RA, Dillon GP (2009). The genome of the blood fluke *Schistosoma mansoni*.. Nature.

[16] Zhou Y, Zheng HJ, Chen YY, Zhang L, Wang K (2009). The *Schistosoma japonicum* genome reveals features of host-parasite interplay.. Nature.

[17] Nakai K, Horton P (1999). PSORT: a program for detecting sorting signals in proteins and predicting their subcellular localization.. Trends Biochem Sci.

[18] Krogh A, Larsson Br, von Heijne G, Sonnhammer ELL (2001). Predicting transmembrane protein topology with a hidden markov model: application to complete genomes.. J Mol Biol.

[19] Fankhauser N, Mäser P (2005). Identification of GPI anchor attachment signals by a Kohonen self-organizing map.. Bioinformatics.

[20] Dosztanyi Z, Csizmok V, Tompa P, Simon I (2005). IUPred: web server for the prediction of intrinsically unstructured regions of proteins based on estimated energy content.. Bioinformatics.

[21] McDonnell AV, Jiang T, Keating AE, Berger B (2006). Paircoil2: improved prediction of coiled coils from sequence.. Bioinformatics.

[22] Kohn WD, Hodges RS (1998). De novo design of alpha-helical coiled coils and bundles: models for the development of protein-design principles.. Trends Biotechnol.

[23] McManus DP, Zhang WB, Li J, Bartley PB (2003). Echinococcosis.. Lancet.

[24] Gottstein B (1992). Molecular and immunological diagnosis of echinococcosis.. Clin Microbiol Rev.

[25] Eckert J, Deplazes P (2004). Biological, epidemiological and clinical aspects of echinococcosis, a zoonosis of increasing concern.. Clin Microbiol Rev.

[26] Müller N, Frei E, Nuñez S, Gottstein B (2007). Improved serodiagnosis of alveolar echinococcosis of humans using an *in vitro* produced *Echinococcus multilocularis* antigen.. Parasitology.

[27] Gottstein B (1985). Purification and characterization of a specific antigen from *Echinococcus multilocularis*.. Parasite Immunol.

[28] Larsen J, Lund O, Nielsen M (2006). Improved method for predicting linear B-cell epitopes.. Immunome Res.

[29] González-Sapienza G, Cachau RE (2003). Identification of critical residues of an immunodominant region of *Echinococcus granulosus* antigen B.. J Biol Chem.

[30] González-Sapienza G, Lorenzo C, Nieto A (2000). Improved immunodiagnosis of cystic hydatid disease by using a synthetic peptide with higher diagnostic value than that of its parent protein, *Echinococcus granulosus* antigen B.. J Clin Microbiol.

[31] González G, Spinelli P, Lorenzo C, Hellman U, Nieto A (2000). Molecular characterization of P-29, a metacestode-specific component of *Echinococcus granulosus* which is immunologically related to, but distinct from, antigen 5.. Mol Biochem Parasitol.

[32] Mühlschlegel F, Frosch P, Castro A, Apfel H, Müller A (1995). Molecular cloning and characterization of an *Echinococcus multilocularis* and *Echinococcus granulosus* stress protein homologous to the mammalian 78 kDa glucose regulated protein.. Mol Biochem Parasitol.

[33] Delunardo F, Ortona E, Margutti P, Perdicchio M, Vacirca D (2010). Identification of a novel 19kDa *Echinococcus granulosus* antigen.. Acta Trop.

[34] Auer H, Stöckl C, Suhendra S, Schneider R (2009). Sensitivity and specificity of new commercial tests for the detection of specific *Echinococcus* antibodies.. Wien Klin Wochensch.

[35] Gomara MJ, Haro I (2007). Synthetic peptides for the immunodiagnosis of human diseases.. Curr Med Chem.

[36] Manocha M, Chitralekha KT, Thakar M, Shashikiran D, Paranjape RS (2003). Comparing modified and plain peptide linked enzyme immunosorbent assay (ELISA) for detection of human immunodeficiency virus type-1 (HIV-1) and type-2 (HIV-2) antibodies.. Immunol Lett.

[37] Fachiroh J, Paramita DK, Hariwiyanto B, Harijadi A, Dahlia HL (2006). Single-assay combination of Epstein-Barr virus (EBV) EBNA1-and viral capsid antigen-p18-derived synthetic peptides for measuring anti-EBV immunoglobulin G (IgG) and IgA antibody levels in sera from nasopharyngeal carcinoma patients: Options for field screening.. J Clin Microbiol.

[38] Shen GM, Behera D, Bhalla M, Nadas A, Laal S (2009). Peptide-Based Antibody Detection for Tuberculosis Diagnosis.. Clin Vaccine Immunol.

[39] Morré SA, Munk C, Persson K, Krüger-Kjaer S, van Dijk R (2002). Comparison of three commercially available peptide-based immunoglobulin G (IgG) and IgA assays to microimmunofluorescence assay for detection of *Chlamydia trachomatis* antibodies.. J Clin Microbiol.

[40] Zhu H, Hu SH, Jona G, Zhu XW, Kreiswirth N (2006). Severe acute respiratory syndrome diagnostics using a coronavirus protein microarray.. Proc Natl Acad Sci U S A.

[41] Hernández M, Beltrán C, García E, Fragoso G, Gevorkian G (2000). Cysticercosis: towards the design of a diagnostic kit based on synthetic peptides.. Immunol Lett.

[42] Van Regenmortel MHV (2001). Antigenicity and immunogenicity of synthetic peptides.. Biologicals.

[43] Andresen H, Grotzinger C, Zarse K, Kreuzer OJ, Ehrentreich-Forster E (2006). Functional peptide microarrays for specific and sensitive antibody diagnostics.. Proteomics.

[44] Ivanov VS, Suvorova ZK, Tchikin LD, Kozhich AT, Ivanov VT (1992). Effective method for synthetic peptide immobilization that increases the sensitivity and specificity of ELISA procedures.. J Immunol Methods.

[45] Ouyang L, Yi XY, Zeng XF, Zhou JC, Wang QL (2003). Partial protection induced by phage library-selected peptides mimicking epitopes of *Schistosoma japonicum*.. Chin Med J.

[46] Hell RCR, Amim P, de Andrade HM, de Avila RAM, Felicori L (2009). Immunodiagnosis of human neurocysticercosis using a synthetic peptide selected by phage-display.. Clin Immunol.

