# Supplementary figures and images for: Serodiagnosis of Echinococcus spp. Infection: Explorative Selection of Diagnostic Antigens by Peptide Microarray
Source: PLoS Negl Trop Dis. 2010 Aug 3;4(8):e771. doi: 10.1371/journal.pntd.0000771 (PMC2914747; doi:10.1371/journal.pntd.0000771)

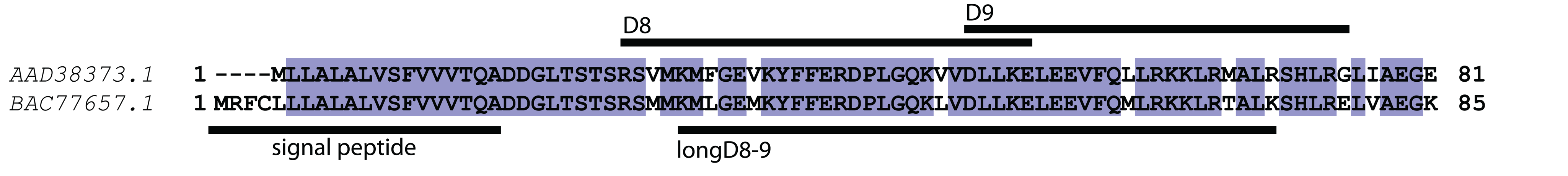

Supplement: Figure S1 — Alignment of E. granulosus and E. multilocularis antigenB8/1 sequences with location of peptide D8, D9 and longD8-9. (0.42 MB TIF) [file pntd.0000771.s001.tif]

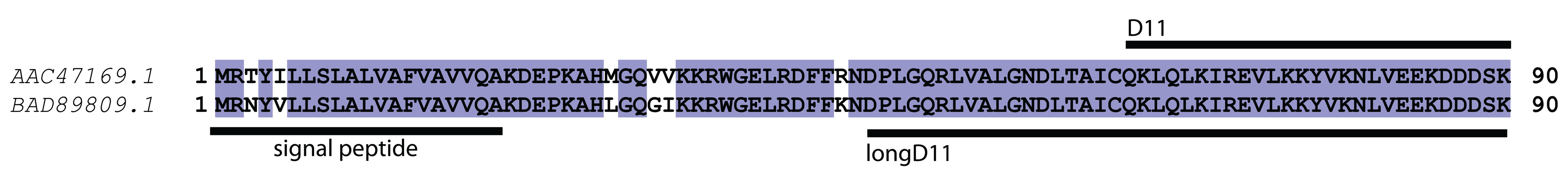

Supplement: Figure S2 — Alignment of E. granulosus and E. multilocularis antigenB8/2 sequences with location of D11 and longD11. (0.44 MB TIF) [file pntd.0000771.s002.tif]
