# Supplementary material for: Serodiagnosis of Echinococcus spp. Infection: Explorative Selection of Diagnostic Antigens by Peptide Microarray
Source: PLoS Negl Trop Dis. 2010 Aug 3;4(8):e771. doi: 10.1371/journal.pntd.0000771 (PMC2914747; doi:10.1371/journal.pntd.0000771)
Supplement: Table S1 — Final set of peptides spotted onto microarray. (0.12 MB DOC) [file pntd.0000771.s003.doc]

Supplementary table 1: Final set of peptides spotted onto microarray.

| Species | Protein | Accession number | Peptide  name | Peptide sequence* | Length (amino acids) | Structure |
| --- | --- | --- | --- | --- | --- | --- |
| E. multilocularis | EmII/3 | AAA50580 | A2 | PIDRLITSKEQYDQTDEQWYERIIAYYKDH | 30 | helical |
| E. multilocularis | EmII/3 | AAA50580 | A3 | EKSKINKRILALCTGNHELYMRRRKSDS | 28 | helical |
| E. multilocularis | EmII/3 | AAA50580 | A4 | KEAERQRLKEERLQRMENEQKLRELRAQ | 28 | CC |
| E. multilocularis | EmII/3 | AAA50580 | D1 | RAQMVEKESDLADMKNKASAYESKIAELE | 29 | CC |
| E. multilocularis | EmII/3 | AAA50580 | D12 | KKEAEKAQAEAELRRMREKHDAKHK | 25 | CC |
| E. multilocularis | EmII/3 | AAA50580 | A5 | NVRRTEESRVTAVSKNETLQTKLANLKME | 29 | CC |
| E. multilocularis | EmII/3 | AAA50580 | A6 | STRDQSKMRDIDRRHEYNVREGNDKYKTLR | 30 | IUR |
| E. multilocularis | EM13 | Q07840 | A7 | SIKEVKNFDSEFENAQKTWYKHYKNVNR | 28 | helical |
| E. multilocularis | EM13 | Q07840 | A8 | HACKTVRSLQVQVQNAKNEPFGTPEQLRK | 29 | helical |
| E. multilocularis | EM13 | Q07840 | A9 | EQLRKIEDKLRKGIMEEEKTRKAYEEALS | 29 | CC |
| E. multilocularis | EM13 | Q07840 | A10 | HTTAYGSNSYDHGSEGATPSDYTS | 24 | IUR |
| E. multilocularis | EM13 | Q07840 | A11 | DELSFNSGDLFEKLEDEDEQGWCKGRKD | 28 | CC |
| E. multilocularis | antigen 6 | AAB61984 | B1 | DAFQKNTEKITTTDKLGTALEQVASQSEK | 29 | helical |
| E. multilocularis | antigen 6 | AAB61984 | B2 | EAQKAKTKLEEVRLDLDSDKTKLKNAKTAE | 30 | CC |
| E. multilocularis | antigen 6 | AAB61984 | B3 | KNAKTAEQKAKWEAEVRKDESDFDRVHQES | 30 | helical |
| E. multilocularis | protoscolex specific coiled-coil protein | CAD44854 | B4 | RQENQRLFEQFCQQIHNIQREKETVRLR | 28 | helical |
| E. multilocularis | protoscolex specific coiled-coil protein | CAD44854 | B5 | EIINLRGEVQQQKRRSGQRTQEHAETIQS | 29 | CC |
| E. multilocularis | protoscolex specific coiled-coil protein | CAD44854 | B6 | EHNAALQQKLDEANQSVTEVSVQMK | 25 | CC |
| E. multilocularis | protoscolex specific coiled-coil protein | CAD44854 | B7 | ESLRAEINHLKEDKATLEKKLQAIMDERDS | 30 | CC |
| E. multilocularis | protoscolex specific coiled-coil protein | CAD44854 | B8 | EDDWWYKHVHLDPSMTGFYEDDEIEIDDAS | 30 | CC |
| E. multilocularis | protoscolex specific coiled-coil protein | CAD44854 | B9 | SDLECNDSGAGGSTEEEFLRSFRTEVAE | 28 | helical |
| E. multilocularis | glucose regulated protein | Q24895 | B10 | DDRAVQKLRREVEKAKRTLSTEHSTMIEID | 30 | helical |
| E. multilocularis | glucose regulated protein | Q24895 | B11 | SAEDKGTGKKSNIVINKETNRLTPEEIER | 29 | IUR |
| E. multilocularis | glucose regulated protein | Q24895 | B12 | EIERMIQDAEKFSDQDKQVKERVEVRNDLE | 30 | IUR |
| E. multilocularis | glucose regulated protein | Q24895 | C1 | QVKDKEKMGGKLSDDEIKTIEDAADEAIK | 29 | IUR |
| E. multilocularis | glucose regulated protein | Q24895 | C2 | AIKWMENNPQAETSDYKKQKANLES | 25 | IUR |
| E. multilocularis | tropomyosin | CAC85552 | C3 | DFEKKEEEMNDWLSKVKNIQTEVDTVQES | 29 | CC |
| E. multilocularis | tropomyosin | CAC85552 | C4 | KLEETEKRATNAEAEVAAMTRRIRLLEED | 29 | CC |
| E. multilocularis | tropomyosin | CAC85552 | C5 | TKLDDASKAAEESERNRKTLETRSISDDER | 30 | CC |
| E. multilocularis | tropomyosin | CAC85552 | C6 | RKYDEAARRLAVTEVDLERAESRLETSESK | 30 | CC |
| E. multilocularis | tropomyosin | CAC85552 | C8 | ERLKTAEQRAAEAERQVSKLQNEVDRLEDE | 30 | CC |
| E. granulosus | HSP70 | Q24789 | C9 | EVKSTAGDTHLGGEDFDSRLVNHFVEEFKR | 30 | helical |
| E. granulosus | HSP70 | Q24789 | C10 | ELCSDLFRSTLDPVEKALRDAKLDKGAVHE | 30 | helical |
| E. granulosus | HSP70 | Q24789 | C11 | SAVDKSTGKQNKITITRDKGRLSKEEIER | 29 | IUR |
| E. granulosus | HSP70 | Q24789 | C12 | KSTVEDEKVKEKIGESDRRRIMEKCEETVK | 30 | IUR |
| E. granulosus | HSP70 | Q24789 | D2 | GNQQAEKEEYEHRQKELESVCNPIIAK | 27 | helical |
| E. granulosus | EG19 antigen | ABI24154 | D3 | EAEAKCLRRPHQRVVKEGEVSKGDEVD | 27 | IUR |
| E. granulosus | EG19 antigen | ABI24154 | D4 | HEVSHEGKQESEDKDADKIAIEGVVRK | 27 | IUR |
| E. granulosus | EG19 antigen | ABI24154 | D5 | AHLGTGKSQHADEKALFYEEEAEDEGEDDE | 30 | IUR |
| E. granulosus | P-29 | AAD53328 | D6 | KAAPQLSKMLTEASDVHQRMATARKNFNSE | 30 | IUR |
| E. granulosus | antigen B8/1 | AAD38373 | D7 | QADDGLTSTSRSVMKMFGEVKYFFERDPLG | 30 | helical |
| E. granulosus | antigen B8/1 | AAD38373 | D8 | RSVMKMFGEVKYFFERDPLGQKVVDLLKE | 29 | helical |
| E. granulosus | antigen B8/1 | AAD38373 | D9 | DLLKELEEVFQLLRKKLRMALRSHLRG | 27 | CC |
| E. granulosus | antigen B8/2 | AAC47169 | D10 | QAKDEPKAHMGQVVKKRWGELRDFFRNDPL | 30 | helical |
| E. granulosus | antigen B8/2 | AAC47169 | D11 | QKLQLKIREVLKKYVKNLVEEKDDDSK | 27 | CC |

* Additionally to the sequence listed, the peptides carried biotin and AHX-spacer at the N-terminus and were amidated at the C-terminus.
